# Supplementary material for: Testing the adaptive hypothesis of lagging-strand encoding in bacterial genomes
Source: Nat Commun. 2022 May 12;13:2628. doi: 10.1038/s41467-022-30000-8 (PMC9098844; doi:10.1038/s41467-022-30000-8)
Supplement: Supplementary file 2 — Description of Additional Supplementary Files [file 41467_2022_30000_MOESM2_ESM.pdf]

Description of Additional Supplementary Files

File Name: Supplementary Data 1
Description: Orthologs identified in this study, with information on gene orientation.
